# Effect of oral sodium bicarbonate supplementation on progression of chronic kidney disease in patients with chronic metabolic acidosis: study protocol for a randomized controlled trial (SoBic-Study)

**DOI:** 10.1186/1745-6215-14-196

**Published:** 2013-07-04

**Authors:** Martina Gaggl, Daniel Cejka, Max Plischke, Georg Heinze, Melanie Fraunschiel, Alice Schmidt, Walter H Hörl, Gere Sunder-Plassmann

**Affiliations:** 1 Department of Medicine III, Division of Nephrology and Dialysis, Medical University of Vienna, Vienna, Austria; 2 Center of Medical Statistics, Informatics and Intelligent Systems, Section of Clinical Biometrics, Medical University of Vienna, Vienna, Austria; 3 Center of Medical Statistics, Informatics and Intelligent Systems, Section for Medical Information Management and Imaging, Medical University of Vienna, Vienna, Austria

**Keywords:** Metabolic acidosis, Chronic kidney disease, Kidney injury, Sodium bicarbonate, Acid–base balance, Acid retention, Acidotic, Progression

## Abstract

**Background:**

Overt chronic metabolic acidosis in patients with chronic kidney disease develops after a drop of glomerular filtration rate to less than approximately 25 mL/min/1.73 m^2^. The pathogenic mechanism seems to be a lack of tubular bicarbonate production, which in healthy individuals neutralizes the acid net production. As shown in several animal and human studies the acidotic milieu alters bone and vitamin D metabolism, induces muscle wasting, and impairs albumin synthesis, aside from a direct alteration of renal tissue by increasing angiotensin II, aldosteron and endothelin kidney levels. Subsequent studies testing various therapeutic approaches in very selected study populations showed that oral supplementation of the lacking bicarbonate halts progression of decline of renal function. However, due to methodological limitations of these studies further investigations are of urgent need to ensure the validity of this therapeutic concept.

**Methods/Design:**

The SoBic-study is a single-center, randomized, controlled, open-label clinical phase IV study performed at the nephrological outpatient service of the Medical University of Vienna. Two-hundred patients classified to CKD stage 3 or 4 with two separate measurements of HCO_3_^-^ of <21 mmol/L will be 1:1 randomized to either receive a high dose of oral sodium bicarbonate with a serum target HCO_3_^-^ level of 24 ± 1 mmol/L or receive a rescue therapy of sodium bicarbonate with a serum target level of 20 ± 1 mmol/L. The follow up will be for two years. The primary outcome is the effect of sodium bicarbonate supplementation on renal function measured by means of estimated glomerular filtration rates (4-variable-MDRD-equation) after two years. Secondary outcomes are change in markers of bone metabolism between groups, death rates between groups, and the number of subjects proceeding to renal replacement therapy across groups. Adverse events, such as worsening of arterial hypertension due to the additional sodium consumption, will be accurately monitored.

**Discussion:**

We hypothesize that sufficiently balanced acid–base homeostasis leads to a reduction of decline of renal function in patients with chronic kidney disease. The concept of an exogenous bicarbonate supplementation to substitute the lacking endogenous bicarbonate has existed for a long time, but has never been investigated sufficiently to state clear treatment guidelines.

**Trial registration:**

EUDRACT Number: 2012-001824-36

## Background

Chronic kidney disease (CKD) occurs in approximately one in ten persons worldwide and represents not only a medical challenge but also a great socioeconomic burden. Major efforts have been made to investigate treatment options aiming to slow progression to end-stage renal disease (ESRD) and, subsequently, to reduce mortality in kidney patients. A renin-angiotensin system blockade with either angiotensin-I-converting enzyme inhibitors or angiotensin-II-receptor antagonists slows the progression of decline in renal function [1-5]. However, further therapeutic interventions have not shown any benefits [6-10]. The comprehensive summery by Fink *et al.* underscores the lack of evidence-based treatment concepts in CKD and even calls screening and monitoring for early-stage CKD into question [11].

Overt chronic metabolic acidosis in CKD patients develops after a drop in glomerular filtration rate (GFR) to less than approximately 25 mL/min/1.73 m^2^. The pathogenic mechanism seems to be a lack of tubular bicarbonate production, which in healthy individuals neutralizes the acid net production. As shown in several animal and human studies the acidotic milieu alters bone and vitamin D metabolism [12], induces muscle wasting [13], and impairs albumin synthesis [14], in addition to other detrimental effects [15,16]. Experiments in rats suggest that even in the earlier stages of CKD with serum bicarbonate levels within normal limits, increased per-nephron acidification occurs to obviate overt metabolic acidosis [17]. Investigations to elucidate the contribution to the progression of CKD itself have provided contradictory results [18-20]. However, there is evidence that the alternative complement system is activated by increased tubular ammonia production [21] and that endothelin, angiotensin II and aldosteron kidney concentrations are increased as a direct result of distal nephron acidification [17,20,22]. Taken together, these alterations induce tubulointerstitial scarring in the long term.

The results of several retrospective studies conducted in different types of populations showed clear associations between acid–base imbalance and increased mortality [23,24]. Above all is the recently published analysis of the CKD registry of the Cleveland Clinic health care provider in 41,749 patients with CKD stage III and IV (estimated (e) GFR of 60 to 15 mL/min/1.73 m^2^, measured by the Chronic Kidney Disease Epidemiology Collaboration equation). Adjusted for several covariates the all-cause mortality for subjects with a low serum bicarbonate level (<23 mmol/L) is 23% higher than for those with a level within the normal range (23 to 32 mmol/L) [25].

The first prospective studies in closely selected study populations [26] using various therapeutic interventions [27] and endpoints revealed beneficial effects. In particular, de Brito-Ashurst *et al.* showed that sodium bicarbonate supplementation in patients with CKD stage IV slows the decline in renal function, although the study comprised some serious methodological limitations [28].

In line with the aforementioned studies we assume that a sufficiently balanced acid–base status leads to a reduction in the decline of renal function in patients with CKD. Bicarbonate is necessary to counterbalance acid net production arising from protein metabolism and acid-rich diets. Thus, the intervention of exogenous bicarbonate supplementation to substitute the lacking endogenous bicarbonate would appear to be rational. This simple concept has existed for a long time, but has never been investigated sufficiently to state clear treatment guidelines for physicians. Furthermore, including patients with both moderate and severe grades of renal impairment represents a novel and comprehensive approach. Thus, conclusions drawn by results of this study are directly applicable to routine care and treatment guidelines.

## Methods/Design

### Trial design

The SoBic study is a single-center, randomized, controlled, stratified, open-label clinical phase IV study and will be performed at the Nephrological outpatient service of the Medical University of Vienna. From the beginning of October 2013, 200 subjects with CKD stage III and IV and chronic metabolic acidosis will be randomized to either receive a high dose of oral sodium bicarbonate with a serum target HCO_3_^-^ level of 24 ± 1 mmol/L or receive a rescue therapy of sodium bicarbonate, if necessary, with a serum target level of 20 ± 1 mmol/L. The follow up period will be 2 years. The ethics committee of the Medical University of Vienna and the Austrian Competent Authority approved the study. The study is registered at the European Union Drug Regulating Authority (EUDRACT Number: 2012-001824-36; https://www.clinicaltrialsregister.eu) and will be conducted in accordance with the current version of the Declaration of Helsinki [29]. All study subjects will be appropriately insured and are required to give written informed consent before any study action is taken. Data will be stored in the Clincase version 2.6.0.33 clinical trial system (Quadratek Data Solutions Ltd, London, United Kingdom. Data structure design is developed by the science support work group of AKIM (Allgemeines Krankenhaus Informations Management) at the Center of Medical Statistics, Informatics and Intelligent Systems. The software provides advanced data management and data preparation tools for statistic evaluation. The web-based electronic case report forms allow efficient user handling and thus, error avoidance. In accordance with the Good Clinical Practice (GCP) guidelines an independent person will periodically perform data monitoring [30]. The study will be reported as suggested by the Consolidated Standards of Reporting Trials (CONSORT) 2010 guidelines [31].

### Participants

The study population will comprise incident and prevalent patients with CKD from the outpatient service of a tertiary care facility. Patients classified as having CKD stage III or IV, and two separate measurements of HCO_3_^-^ of <21 mmol/L (at least 1 day apart) will be included in the study. Patients fulfilling the inclusion criteria, who are already receiving alkali treatment, can be included after a wash-out phase of 4 weeks. The inclusion criteria are age over 18 years, renal function (measured by eGFR calculated by the four-variable Modification of Diet in Renal Disease (MDRD) study equation) between 60 and 15 mL/min/1.73 m^2^, venous serum HCO_3_^-^ of <21 mmol/L on two consecutive measurements (at least 1 day apart), and for the patient to be in a stable clinical condition. Patients with malignant disease or within 5 years of successful treatment of malignant disease (except dermal malignancies and carcinoma *in situ* of the cervix declared to be cured), and patients with morbid obesity (body mass index >40 kg/m^2^), chronic inflammation (C reactive protein >10 mg/dL) or taking immunosuppressive therapy of any kind, poorly controlled blood pressure (>150/90 mmHg despite the use of four agents), overt congestive heart failure, or known peanut or soy allergy, will be excluded from the study.

With respect to concomitant medication, various drugs are known to induce metabolic acidosis [32]. However, most relevant with regard to the planned study are the phosphate binder sevelamer hydrochloride and potassium-sparing diuretics. Other agents known to neutralize metabolic acidosis, such as calcium citrate, sodium citrate and calcium carbonate are not allowed.

### Laboratory measurements

Serum creatinine will be determined by means of the Jaffe method (reference range <1.2 mg/dL, mg/dL × 88.4 = μmol/L). Bicarbonate measurements will be performed in venous blood samples and determined by an ABL 700 Copenhagen Blood Gas Analyzer (Radiometer Copenhagen, Copenhagen, Denmark). Markers of bone metabolism (bone alkaline phosphatase, osteocalcin, c-telopeptide pyridinoline crosslinks of type I collagen, procollagen type I N-propeptide) will be measured as detailed in Cejka *et al.*[33]. All laboratory measurements, including urinalyses, will be performed in an ISO 9001–2008 certified and ISO 15189-accredited clinical laboratory at the Medical University Vienna [http://www.kimcl.at].

### Randomization and study visits

**Figure 1:**
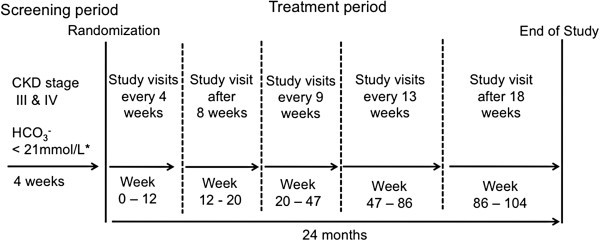
Study design. ^*^Two consecutive measurements at least 1 day apart. CKD, chronic kidney disease.

Subjects will attend the clinic for a regular visit and blood draw. After a careful review of their medical history patients who fulfill the inclusion criteria will be contacted and informed about the study protocol. Patients who agree to participate in the study will be invited to a further blood work-up to ensure that metabolic acidosis is chronic rather than a one-time imbalance of the acid–base status. After obtaining written informed consent (day 0) each patient will be randomized 1:1 either to the treatment group or the control group (Figure 1). Subjects will be stratified for age >60 years and ≤60 years [34], presence of diabetes mellitus (yes/no) [35], severe chronic metabolic acidosis (serum HCO_3_^-^ <18 mmol/L) versus moderate metabolic acidosis (serum HCO_3_^-^ ≥18 mmol/L), and for previous alkali treatment (yes/no). Previous alkali treatment is defined as being continued for longer than 6 months (>25% of the treatment period of the planned study) and serum HCO_3_^-^ ≥20 mmol/L on more than 75% of measurements. Stratified randomization will be carried out using the web-based randomizer program provided by the Medical University of Vienna [https://www.meduniwien.ac.at/randomizer/web/login.php]. We use the minimization method with a preferred treatment probability of 0.9, and a combination depth of 2.0, to guarantee that treatment allocation is balanced in all levels of the stratification factors, as well as in all pairwise combinations of stratification factors. After randomization, subjects will receive either the high dose of sodium bicarbonate or, if necessary, a low-dose rescue therapy with sodium bicarbonate.

Each study visit consists of a fasting blood draw (last food intake including the study medication should be at least 8 hours before) and urinalysis, measurement of vital signs, and assessment of changes in concomitant medication and adverse events (Table 1). At the beginning of the study a physical examination will be done and body weight and height will be assessed. Study visits will be performed at weeks 4, 8, 12, 20, 29, 38, 47, 60, 73, 86, and 104.

**Table 1:** Visit and assessment schedule

	**Screening period (4 weeks)**							**Treatment period (0 to 24 months)**					
		**Day 0**	**Week 4**	**Week 8**	**Week 12**	**Week 20**	**Week 29**	**Week 38**	**Week 47**	**Week 60**	**Week 73**	**Week 86**	**Week 104**
			**(± 2 days)**	**(± 2 days)**	**(± 2 days)**	**(± 5 days)**	**(± 5 days)**	**(± 5 days)**	**(± 5 days)**	**(± 10 days)**	**(± 10 days)**	**(± 10 days)**	**(± 10 days)**
**Visit**	1	2	3	4	5	6	7	8	9	10	11	12	13
**Informed consent**		x											
**Inclusion/exclusion criteria**	x	x											
**Randomization**		x											
**Medical history**		x											x
**Concomitant medication**		x	x	x	x	x	x	x	x	x	x	x	x
**Physical examination**		x											x
**Body weight and height**		x											x
**Vital signs (BP, PR)**		x	x	x	x	x	x	x	x	x	x	x	x
**Laboratory test**	x	x	x	x	x	x	x	x	x	x	x	x	x
**Laboratory test: bone markers**		x				x							x
**Biobank sample**		x				x			x		x		x
**Urinalysis**	x	x	x	x	x	x	x	x	x	x	x	x	x
**24-hour urine collection**	x^a^	x^a^	x			x			x				x
**Urine pregnancy test**^**b**^		x	x	x	x	x	x	x	x	x	x	x	x
**Adverse events**			x	x	x	x	x	x	x	x	x	x	

^**a**^To be performed either at screening visit 1 or screening visit 2. prior to randomization; ^**b**^for females of childbearing age only. BP, blood pressure; PR, pulse rate.

### Intervention

The initial and the maintenance dose for the intervention group will be determined based on the baseline HCO_3_^-^ level and the levels measured during follow up visits. Based on pilot data we expect the daily dose for the intervention group to vary between two and six capsules of Nephrotrans® 840 mg (1,680 to 5,040 mg sodium bicarbonate per day). In the case of HCO_3_^-^ lower than 19 mmol/L at the baseline visit, or during follow up, subjects randomized to the control group will receive rescue therapy. We expect the daily dose to be zero to three capsules of Nephrotrans® 840 mg (0 to 2,520 mg sodium bicarbonate per day) for the control group. After each study visit the study drug can be down- and up-titrated according to the actual HCO_3_^-^ level and the respective target value, as pre-specified at randomization, by means of the suggested treatment algorithm (Table 2A and B). However, due to intra-individual differences in drug response the algorithm is not obligatory and might be modified if necessary to reach the target HCO_3_^-^ level for respective subjects.

**Table 2:** Study drug up- and down-titration

	**Daily sodium bicarbonate (Nephrotrans® 840 mg) dosage**	
**(A) Baseline treatment algorithm**	Investigational group	Control group
Mean HCO_3_^-^ of two measurements		
<18 mmol/L	5,040 mg (2 capsules TID)	2,520 mg (1 capsule TID)
18 to 19 mmol/L	4,200 mg (2 capsules BID, 1 capsule QD)	1,680 mg (1 capsule BID)
19.1 to 20.0 mmol/L	2,520 mg (1 capsule TID)	Monitor
>20 mmol/L	1,680 mg (1 capsule BID)	
**(B) Follow-up visit treatment algorithm**	Investigational group and control group	
Difference to target HCO_3_^-^ level		
−1 mmol/L	Add 1,680 mg to the previous daily dosage (+1 capsule BID)	
−2 mmol/L	Add 3,360 mg to the previous daily dosage (+2 capsules BID)	
≤ −3 mmol/L	Add 5,040 mg to the previous daily dosage (+2 capsules TID)	
+1 mmol/L	Subduct 1,680 mg from the previous daily dosage (−2 capsules)	
+2 mmol/L	Subduct 3,360 mg from the previous daily dosage (−4 capsules)	
≥ +3 mmol/L	Subduct 5,040 mg from the previous daily dosage (−6 capsules)	

QD, every day; BID, twice daily; TID, trice daily.

### Outcome parameters

The primary outcome is renal function and will be measured by means of eGFR, calculated according to the 4-variable-MDRD Study equation [36].

Secondary outcomes are death, and the need for renal replacement therapy (RRT) (patients with renal transplantation, hemodialysis, peritoneal dialysis, and subjects who are in need of RRT, but personally elect not to start it). The need for RRT will be clinically evaluated, including monitoring of uremic symptoms (nausea and/or vomiting, fatigue, deterioration in nutritional status and significant weight loss, neuropathy, encephalopathy, or psychic disturbances), blood and fluid disturbances (volume overload, malignant hypertension, anemia, bleeding diathesis, pleuritis and/or pericarditis), and significant electrolyte imbalances. A further secondary outcome is the percent change in markers of bone metabolism (bone alkaline phosphatase, osteocalcin, c-telopeptide pyridinoline crosslinks of type I collagen, and procollagen type I N-propeptide) across groups.

### Sample size

**Figure 2:**
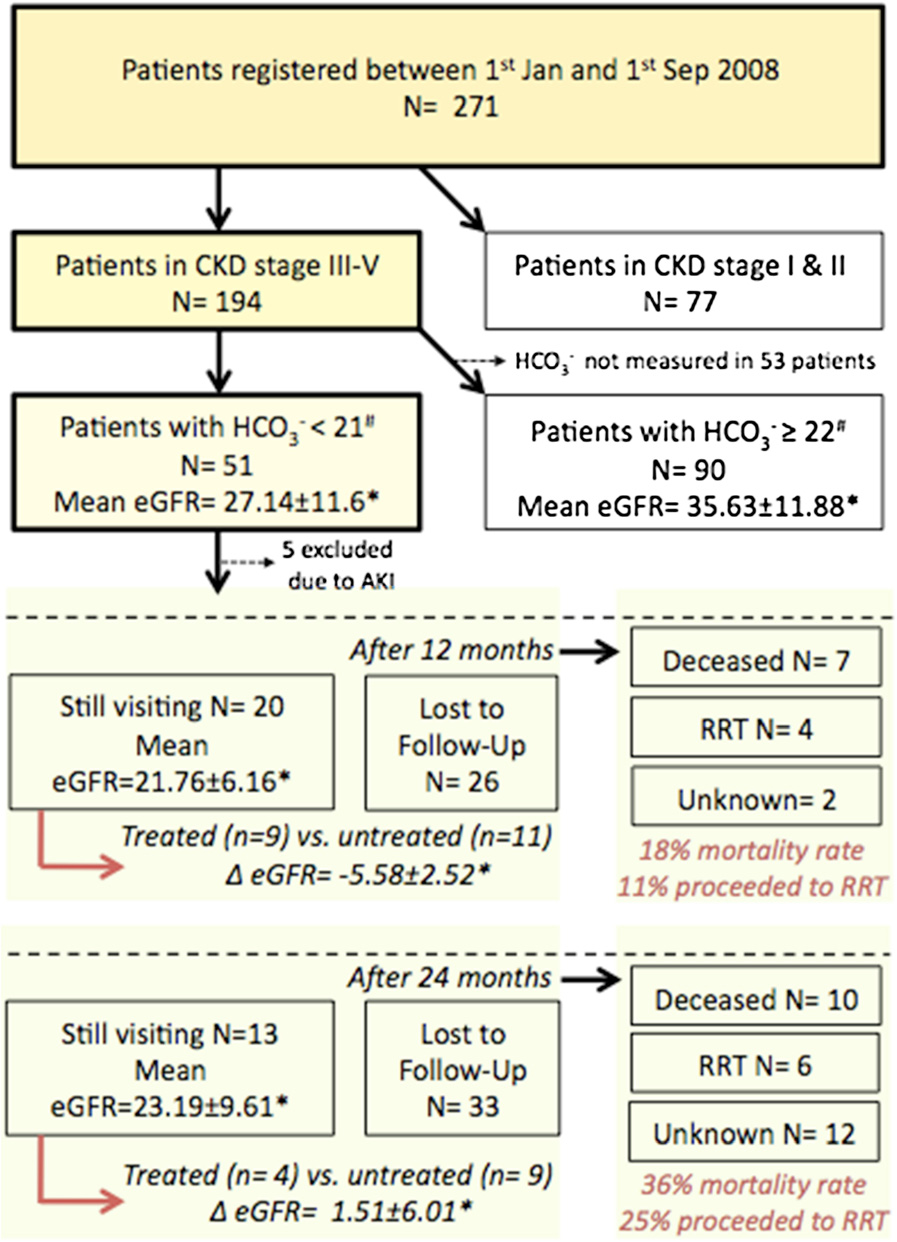
**Cohort analyzed to calculate the sample size.** CKD, chronic kidney disease; eGFR, estimated glomerular filtration rate.

Assuming equal baseline levels of eGFR between the two study groups, a clinically relevant difference of 5 ml in eGFR after 2 years, and an estimated standard deviation of 12 ml for the change in eGFR after 2 years (pilot data, Figure 2), an independent sample *t*-test with a sample size of 2 × 100 patients will give 80% power to reach statistical significance at a two-sided significance level of 5%.

### Statistical methods

The intention-to-treat population will be used for statistical analysis. The primary analysis focuses on the estimated treatment effect at 2 years (difference between the two groups in eGFR after two years), which will be provided as the point estimate at the expected difference, 95% confidence interval and *P*-value. Mortality of patients or other reasons for dropout will be addressed by jointly modeling longitudinal eGFR and time to death using an appropriate joint model for longitudinal and time-to-event outcome [37]. In the longitudinal part of this model, a linear mixed model will be used with the repeated eGFR measurements as outcome values, and including as fixed factors treatment group, all stratification criteria (age, diabetes, severity of acidosis, previous alkali treatment), the eGFR baseline value and time since start of treatment, and a random intercept. An interaction effect (product term) of the treatment group and the time since start of treatment will also be included. Graphical diagnostic tools (residual plots) will be used to confirm the adequacy of model assumptions. A secondary outcome is time to death, compared between the treatment groups, which will also be estimated by the joint modeling approach. The time up to the point of need of RRT will be compared between the two treatment groups, accounting for death as a competing risk, by modeling the cumulative incidence function [38], including treatment group and the covariates age, diabetes, severity of acidosis and baseline eGFR. Percent change (from baseline to week 20 and baseline to week 104, respectively) in markers of bone metabolism will be analyzed by analysis of covariance, to adjust for sex, age, change in renal function, and baseline values of the bone markers. The incidence of serious adverse events will be reported and compared between groups using the chi-squared test. Baseline parameters (etiology of CKD, relevant medical disease, age, gender, concomitant medication, blood pressure, number of antihypertensive medications, and laboratory measurements) will be analyzed descriptively for the interventional and control group, respectively. SAS (Version 9.3, SAS Institute Inc., Cary, NC, USA, 2011) will be used for data management and analysis, and R 2.12.2/package JM [37] will be used for the main outcome analysis.

## Discussion

In 1931 Lyon *et al.* described for the first time the simple approach of compensating metabolic acid excess in a series of 17 patients with chronic nephritis by providing an alkaline diet or substituting alkaline salts [39]. In 2012 the question of whether or not systematic treatment of metabolic acidosis in CKD is indicated is still to be addressed by the scientific community, due to the paucity of acceptable evidence [40-42].

Indeed, patients with CKD comprise a difficult study cohort in terms of sample size calculation and accurate methodological planning, due to severe comorbidities and increased mortality [25,43-47]. De Brito-Ashurst *et al.*[28] tried to show that correcting metabolic acidosis stabilizes renal function in patients with CKD stage IV. In the open-label study the control group had a significantly greater decline in creatinine clearance (5.93 mL/min) compared to the treatment group (1.88 mL/min) after 2 years. Furthermore, subjects in the treatment group presented with significantly slower progression of CKD (defined as creatinine clearance loss >3 mL/min/year) (9% versus 45% in the control group). However, critical issues concerning the study methodology are that first, rapid progression and development of ESRD are competing endpoints, and second, the way this was handled in the data analysis is unclear. They stated that ‘the rate of decline of creatinine clearance was similar between the two groups up to 12 months of follow-up because of dropout of 17 patients of the control group who had rapid decline and reached ESRD between 6 and 12 months’. However, only 15 dropouts are depicted in the respective Kaplan-Meier curve (Figure three in de Brito-Ashurst *et al.*[28]) between months 6 and 12. This might be the source of confounding and can be avoided by using a more appropriate competing endpoint analysis [48]. Further, the need of that additional endpoint is questionable, because subjects with rapid decline in renal function essentially reach ESRD at some point in time. Also, the control group was treated with ‘standard care’ with no further explanation whether that protocol included sodium bicarbonate supplementation. If this was not the case, the administration of placebo in the control group might have been indicated.

Mahajan *et al.*[26] recently compared the effect of sodium bicarbonate, sodium chloride and placebo on GFR in patients with early hypertensive nephropathy over 5 years. This was a randomized blinded trial in patients with CKD stage II, hypertension and albuminuria (200 to 2,000 mg/g creatinine), but normal plasma total carbon dioxide levels (equivalent to serum bicarbonate concentrations). Although the study population showed no biochemical signs of metabolic acidosis the supplementation of sodium bicarbonate was beneficial in terms of renal function: the rate of change of eGFR in the sodium bicarbonate group was −1.47 ± 0.19 compared to −2.13 ± 0.19 (*P* < 0.014) in the placebo group and −2.05 ± 0.19 mL/min/1.73 m^2^ per year (*P* < 0.029) in the sodium chloride group. This gives good evidence that sodium bicarbonate supplementation provides positive effects on renal function on the one hand, but on the other only for that very selected cohort of patients with well-preserved renal function and albuminuria. Unfortunately, one cannot conclude that in patients with advanced renal injury and low GFR the therapeutic intervention would lead to the same outcome.

Another study, although designed to investigate the mechanism of preventing renal injury by oral sodium citrate, showed that treating metabolic acidosis in patients with CKD slows the decline of renal function (sodium citrate versus control: eGFR −1.60 ± 0.13 versus −3.79 ± 0.30 mL/min/1.73 m^2^ per year, *P* < 0.0001) [27]. We refrain from using this agent to treat metabolic acidosis in our study patients due to the known increased aluminum absorption in combination with sodium citrate [49].

Furthermore, studies in animals and humans suggest that an acid-reduced diet may improve kidney function equally to supplementation with bicarbonate by means of tablets [50]. However, base-inducing diets predominantly include vegetables and fruits, which contain high amounts of potassium. In the long term, however, subjects with preserved renal function, who are not prone to hyperkalemia, might benefit tremendously from base-rich diets over the commonly preferred acid-inducing diet of our society [51,52].

With regard to the presented study protocol we opted for an open-label approach, predominantly because to some extent sodium bicarbonate is included in standard routine care. Withdrawing that therapeutic intervention from study subjects could be deemed unethical. Aside from ethical reasons, the dose of the study drug in the interventional group is not fixed and administering an average amount of placebo tablets to the control group is also questionable. Moreover, given the relatively narrow range of suggested optimal HCO_3_^-^ levels and the distinct between-subject difference in response to treatment, blood levels have to be maintained regularly. Blinding of these laboratory results would have caused a disproportionately high amount of effort and cost compared to its benefits.

With regard to the intervention itself, sodium bicarbonate supplementation represents a simple therapeutic maneuver to restore acid–base balance in CKD patients. Few adverse events are reported in combination with oral sodium bicarbonate supplementation. Abdominal bloating would appear to be innocuous as it can be avoided by dividing the daily dosage into several smaller portions.

A mentionable concern is the additional sodium consumption evoked by the study medication. For each 100 mg of sodium bicarbonate the additional sodium intake is approximately 27.4 mg. Dietary sodium intake is known to negatively influence arterial hypertension, therefore, blood pressure should be carefully monitored and if necessary, treated with antihypertensive agents. Notably, none of the studies performed in humans have noticed worsening of arterial hypertension in relation to the sodium bicarbonate supplementation. Nevertheless, this adverse event is reported for animal experiments [18]. However, former investigations suggested that the blood pressure-elevating effect is led by chloride and not by sodium. Although sodium retention and weight gain was similar across study groups an increase in arterial blood pressure could not be detected in subjects ingesting additional dietary sodium bound to an alternative anion than chloride [53-55]. In addition, there is a theoretical concern that long-term sodium bicarbonate intake may favor the development of calcium phosphate- and magnesium phosphate stones in the urinary tract, but so far this has not been observed in humans.

A single study was performed in cultured cells and uremic rats to investigate whether sodium bicarbonate supplementation affects the pathologic calcification of blood vessels. De Solis *et al.* reported that acid media prevented calcification of cultured vascular smooth muscle cells, and that uremic rats treated with sodium bicarbonate had a significantly higher aortic calcification index [56]. Given all the evidence of positive alteration by the supplementation of sodium bicarbonate, these concerns need to be kept in mind, and blood pressure will be closely monitored during the follow up period.

Taken together, previous studies, including observational and animal studies, give confidence that simply supplementing sodium bicarbonate in patients with CKD of any degree could positively alter decline in renal function. Possible adverse events are within the acceptable ratio of benefit to risk, however, adverse events will be followed closely in the study participants.

In summary, we believe that a clinical investigation to elucidate the effect of sodium bicarbonate supplementation in patients with metabolic acidosis and advanced CKD is of urgent need to develop a proper treatment strategy for those patients. This would not only improve the prognosis for each individual patient, but also help to decrease health care expenses, as sodium bicarbonate represents a relatively cheap treatment option.

## Trial status

Recruitment is planned to start in October 2013.

## Abbreviations

AKIM: Allgemeines Krankenhaus informations management; CKD: Ahronic kidney disease; CONSORT: Consolidated standards of reporting trials; eGFR: Estimated GFR; ESRD: End-stage renal disease; EUDRACT: European union drug regulating authority; GFR: Glomerular filtration rate; GCP: Good clinical practice; MDRD: Modification of diet in renal disease; RRT: Renal replacement therapy.

## Competing interests

The authors declare that they have no competing interests.

## Authors’ contributions

MG and GSP designed the study, wrote the study protocol and manuscript, organized the funding, and will conduct the study. GH designed the study with regard to the statistical methodology and will perform randomization and stratification procedures and will perform the statistical analysis. MF programmed the electronic case report forms and will regularly maintain the database. DC, WHH, MP and AS contributed to the planning of the study and critically revised the manuscript. All authors read and approved the final manuscript.

## Authors’ information

We are saddened by the sudden passing of our department head and teacher, Prof. DDr. Walter H. Hörl FRCP, whom we want to dedicate this manuscript.

## Funding

Study medication will be provided by the manufacturer MEDICE Pharma Pütter GmbH & Co. KG without financial compensation.

## References

[B1] Parving HH Lehnert H Brochner-Mortensen J Gomis R Andersen S Arner P The effect of irbesartan on the development of diabetic nephropathy in patients with type 2 diabetes N Engl J Med 2001 345 870 878 10.1056/NEJMoa011489 11565519

[B2] Brenner BM Cooper ME de Zeeuw D Keane WF Mitch WE Parving HH Remuzzi G Snapinn SM Zhang Z Shahinfar S Effects of losartan on renal and cardiovascular outcomes in patients with type 2 diabetes and nephropathy N Engl J Med 2001 345 861 869 10.1056/NEJMoa011161 11565518

[B3] Ruggenenti P Perna A Mosconi L Matalone M Garini G Salvadori M Zoccali C Scolari F Maggiore Q Tognoni G Remuzzi G Randomised placebo-controlled trial of effect of ramipril on decline in glomerular filtration rate and risk of terminal renal failure in proteinuric, non-diabetic nephropathy. The GISEN Group (Gruppo Italiano di Studi Epidemiologici in Nefrologia) Lancet 1997 349 1857 1863 9217756

[B4] Ruggenenti P Perna A Gherardi G Garini G Zoccali C Salvadori M Scolari F Schena FP Remuzzi G Renoprotective properties of ACE-inhibition in non-diabetic nephropathies with non-nephrotic proteinuria Lancet 1999 354 359 364 10.1016/S0140-6736(98)10363-X 10437863

[B5] Hostetter TH Prevention of end-stage renal disease due to type 2 diabetes N Engl J Med 2001 345 910 912 10.1056/NEJM200109203451209 11565525

[B6] Appel LJ Wright JT Jr Greene T Agodoa LY Astor BC Bakris GL Cleveland WH Charleston J Contreras G Faulkner ML Gabbai FB Gassman JJ Hebert LA Jamerson KA Kopple JD Kusek JW Lash JP Lea JP Lewis JB Lipkowitz MS Massry SG Miller ER Norris K Phillips RA Pogue VA Randall OS Rostand SG Smogorzewski MJ Toto RD Wang X Intensive blood-pressure control in hypertensive chronic kidney disease N Engl J Med 2010 363 918 929 10.1056/NEJMoa0910975 20818902 PMC3662974

[B7] Mann JF Schmieder RE McQueen M Dyal L Schumacher H Pogue J Wang X Maggioni A Budaj A Chaithiraphan S Dickstein K Keltai M Metsarinne K Oto A Parkhomenko A Piegas LS Svendsen TL Teo KK Yusuf S Renal outcomes with telmisartan, ramipril, or both, in people at high vascular risk (the ONTARGET study): a multicentre, randomised, double-blind, controlled trial Lancet 2008 372 547 553 10.1016/S0140-6736(08)61236-2 18707986

[B8] Tobe SW Clase CM Gao P McQueen M Grosshennig A Wang X Teo KK Yusuf S Mann JF Cardiovascular and renal outcomes with telmisartan, ramipril, or both in people at high renal risk: results from the ONTARGET and TRANSCEND studies Circulation 2011 123 1098 1107 10.1161/CIRCULATIONAHA.110.964171 21357827

[B9] Ruggenenti P Perna A Tonelli M Loriga G Motterlini N Rubis N Ledda F Rota S Jr Satta A Granata A Battaglia G Cambareri F David S Gaspari F Stucchi N Carminati S Ene-Iordache B Cravedi P Remuzzi G Effects of add-on fluvastatin therapy in patients with chronic proteinuric nephropathy on dual renin-angiotensin system blockade: the ESPLANADE trial Clin J Am Soc Nephrol 2010 5 1928 1938 10.2215/CJN.03380410 20671225 PMC3001777

[B10] Klahr S AS L Beck GJ Caggiula AW Hunsicker L Kusek JW Striker G The effects of dietary protein restriction and blood-pressure control on the progression of chronic renal disease. Modification of diet in renal disease study group N Engl J Med 1994 330 877 884 10.1056/NEJM199403313301301 8114857

[B11] Fink HA Ishani A Taylor BC Greer NL MacDonald R Rossini D Sadiq S Lankireddy S Kane RL Wilt TJ Screening for, monitoring, and treatment of chronic kidney disease stages 1 to 3: a systematic review for the U.S. Preventive services task force and for an American College of Physicians clinical practice guideline Ann Intern Med 2012 156 570 581 10.7326/0003-4819-156-8-201204170-00008 22508734

[B12] Kopple JD Kalantar-Zadeh K Mehrotra R Risks of chronic metabolic acidosis in patients with chronic kidney disease Kidney Int 2005 67 Suppl 95 21 27 10.1111/j.1523-1755.2005.09503.x 15882309

[B13] Lim VS Yarasheski KE Flanigan MJ The effect of uraemia, acidosis, and dialysis treatment on protein metabolism: a longitudinal leucine kinetic study Nephrol Dial Transplant 1998 13 1723 1730 10.1093/ndt/13.7.1723 9681719

[B14] Movilli E Zani R Carli O Sangalli L Pola A Camerini C Cancarini GC Scolari F Feller P Maiorca R Correction of metabolic acidosis increases serum albumin concentrations and decreases kinetically evaluated protein intake in haemodialysis patients: a prospective study Nephrol Dial Transplant 1998 13 1719 1722 10.1093/ndt/13.7.1719 9681718

[B15] Kraut JA Madias NE Consequences and therapy of the metabolic acidosis of chronic kidney disease Pediatr Nephrol 2011 26 19 28 10.1007/s00467-010-1564-4 20526632 PMC2991191

[B16] Kraut JA Kurtz I Metabolic acidosis of CKD: diagnosis, clinical characteristics, and treatment Am J Kidney Dis 2005 45 978 993 10.1053/j.ajkd.2005.03.003 15957126

[B17] Wesson DE Jo CH Simoni J Angiotensin II receptors mediate increased distal nephron acidification caused by acid retention Kidney Int 2012 82 1184 1194 10.1038/ki.2012.267 22832514

[B18] Gadola L Noboa O Marquez MN Rodriguez MJ Nin N Boggia J Ferreiro A Garcia S Ortega V Musto ML Ponte P Sesser P Pizarrosa C Ravaglio S Vallega A Calcium citrate ameliorates the progression of chronic renal injury Kidney Int 2004 65 1224 1230 10.1111/j.1523-1755.2004.00496.x 15086461

[B19] Jara A Felsenfeld AJ Bover J Kleeman CR Chronic metabolic acidosis in azotemic rats on a high-phosphate diet halts the progression of renal disease Kidney Int 2000 58 1023 1032 10.1046/j.1523-1755.2000.00260.x 10972667

[B20] Wesson DE Simoni J Acid retention during kidney failure induces endothelin and aldosterone production which lead to progressive GFR decline, a situation ameliorated by alkali diet Kidney Int 2010 78 1128 1135 10.1038/ki.2010.348 20861823

[B21] Nath KA Hostetter MK Hostetter TH Pathophysiology of chronic tubulo-interstitial disease in rats. Interactions of dietary acid load, ammonia, and complement component C3 J Clin Invest 1985 76 667 675 10.1172/JCI112020 2993363 PMC423874

[B22] Phisitkul S Hacker C Simoni J Tran RM Wesson DE Dietary protein causes a decline in the glomerular filtration rate of the remnant kidney mediated by metabolic acidosis and endothelin receptors Kidney Int 2008 73 192 199 10.1038/sj.ki.5002647 17978813

[B23] Kovesdy CP Anderson JE Kalantar-Zadeh K Association of serum bicarbonate levels with mortality in patients with non-dialysis-dependent CKD Nephrol Dial Transplant 2009 24 1232 1237 19015169 10.1093/ndt/gfn633 PMC2721428

[B24] Shah SN Abramowitz M Hostetter TH Melamed ML Serum bicarbonate levels and the progression of kidney disease: a cohort study Am J Kidney Dis 2009 54 270 277 10.1053/j.ajkd.2009.02.014 19394734 PMC4354889

[B25] Navaneethan SD Schold JD Arrigain S Jolly SE Wehbe E Raina R Simon JF Srinivas TR Jain A Schreiber MJ Jr Nally JV Jr Serum Bicarbonate and Mortality in Stage 3 and Stage 4 Chronic Kidney Disease Clin J Am Soc Nephrol 2011 6 2395 2402 10.2215/CJN.03730411 21885787 PMC3359558

[B26] Mahajan A Simoni J Sheather SJ Broglio KR Rajab MH Wesson DE Daily oral sodium bicarbonate preserves glomerular filtration rate by slowing its decline in early hypertensive nephropathy Kidney Int 2010 78 303 309 10.1038/ki.2010.129 20445497

[B27] Phisitkul S Khanna A Simoni J Broglio K Sheather S Rajab MH Wesson DE Amelioration of metabolic acidosis in patients with low GFR reduced kidney endothelin production and kidney injury, and better preserved GFR Kidney Int 2010 77 617 623 10.1038/ki.2009.519 20072112

[B28] de Brito-Ashurst I Varagunam M Raftery MJ Yaqoob MM Bicarbonate supplementation slows progression of CKD and improves nutritional status J Am Soc Nephrol 2009 20 2075 2084 10.1681/ASN.2008111205 19608703 PMC2736774

[B29] WMA WMA declaration of Helsinki - Ethical principles for medical research involving human subjects 2008 http://www.wma.net/en/30publications/10policies/b3/index.html 19886379

[B30] EMEA Guideline for good clinical practice ICH topic E 2002, 6, Step 5. http://www.ema.europa.eu/docs/en_GB/document_library/Scientific_guideline/2009/09/WC500002874.pdf

[B31] Moher D Hopewell S Schulz KF Montori V Gotzsche PC Devereaux PJ Elbourne D Egger M Altman DG CONSORT 2010 explanation and elaboration: updated guidelines for reporting parallel group randomised trials J Clin Epidemiol 2010 2010 e1 e37 20346624 10.1016/j.jclinepi.2010.03.004

[B32] Liamis G Milionis HJ Elisaf M Pharmacologically-induced metabolic acidosis: a review Drug Saf 2010 33 371 391 10.2165/11533790-000000000-00000 20397738

[B33] Cejka D Jager-Lansky A Kieweg H Weber M Bieglmayer C Haider DG Diarra D Patsch JM Kainberger F Bohle B Haas M Sclerostin serum levels correlate positively with bone mineral density and microarchitecture in haemodialysis patients Nephrol Dial Transplant 2012 27 226 230 10.1093/ndt/gfr270 21613383

[B34] Frassetto LA Morris RC Jr Sebastian A Effect of age on blood acid–base composition in adult humans: role of age-related renal functional decline Am J Physiol 1996 271 F1114 F1122 8997384 10.1152/ajprenal.1996.271.6.F1114

[B35] Caravaca F Arrobas M Pizarro JL Esparrago JF Metabolic acidosis in advanced renal failure: differences between diabetic and nondiabetic patients Am J Kidney Dis 1999 33 892 898 10.1016/S0272-6386(99)70422-1 10213645

[B36] Manjunath G Sarnak MJ Levey AS Prediction equations to estimate glomerular filtration rate: an update Curr Opin Nephrol Hypertens 2001 10 785 792 10.1097/00041552-200111000-00009 11706306

[B37] Rizopulos DJ An R Package for the joint modelling of longitudinal and time-to-event data J Stat Software 2010 35 1 33

[B38] Fine J Gray R A Proportional hazards model for the subdistribution of a competing risk J Am Stat Assoc 1999 94 496 509 10.1080/01621459.1999.10474144

[B39] Lyon MD Dunlop DM Stewart CP The alkaline treatment of chronic nephritis Lancet 1931 2 1009 1013

[B40] Sahni V Rosa RM Batlle D Potential benefits of alkali therapy to prevent GFR loss: time for a palatable ‘solution’ for the management of CKD Kidney Int 2010 78 1065 1067 10.1038/ki.2010.364 21076447

[B41] Abboud H Henrich WL Clinical practice. Stage IV chronic kidney disease N Engl J Med 2010 362 56 65 10.1056/NEJMcp0906797 20054047

[B42] Frassetto LA Hsu CY Metabolic acidosis and progression of chronic kidney disease J Am Soc Nephrol 2009 20 1869 1870 10.1681/ASN.2009070710 19696222

[B43] John R Webb M Young A Unreferred chronic kidney disease: a longitudinal study Am J Kidney Dis 2004 43 825 835 10.1053/j.ajkd.2003.12.046 15112173

[B44] Conway B Webster A Ramsay G Morgan N Neary J Whitworth C Harty J Predicting mortality and uptake of renal replacement therapy in patients with stage 4 chronic kidney disease Nephrol Dial Transplant 2009 24 1930 1937 10.1093/ndt/gfn772 19181760

[B45] Levin A Djurdjev O Beaulieu M Er L Variability and risk factors for kidney disease progression and death following attainment of stage 4 CKD in a referred cohort Am J Kidney Dis 2008 52 661 671 10.1053/j.ajkd.2008.06.023 18805347

[B46] Holland DC Lam M Predictors of hospitalization and death among pre-dialysis patients: a retrospective cohort study Nephrol Dial Transplant 2000 15 650 658 10.1093/ndt/15.5.650 10809806

[B47] Go AS Chertow GM Fan D McCulloch CE Hsu CY Chronic kidney disease and the risks of death, cardiovascular events, and hospitalization N Engl J Med 2004 351 1296 1305 10.1056/NEJMoa041031 15385656

[B48] Agarwal R Bunaye Z Bekele DM Light RP Competing risk factor analysis of end-stage renal disease and mortality in chronic kidney disease Am J Nephrol 2008 28 569 575 10.1159/000115291 18239383

[B49] Nolan CR Califano JR Butzin CA Influence of calcium acetate or calcium citrate on intestinal aluminum absorption Kidney Int 1990 38 937 941 10.1038/ki.1990.294 2266679

[B50] Goraya N Simoni J Jo C Wesson DE Dietary acid reduction with fruits and vegetables or bicarbonate attenuates kidney injury in patients with a moderately reduced glomerular filtration rate due to hypertensive nephropathy Kidney Int 2012 81 86 93 10.1038/ki.2011.313 21881553

[B51] Krishnamurthy VM Wei G Baird BC Murtaugh M Chonchol MB Raphael KL Greene T Beddhu S High dietary fiber intake is associated with decreased inflammation and all-cause mortality in patients with chronic kidney disease Kidney Int 2012 81 300 306 10.1038/ki.2011.355 22012132 PMC4704855

[B52] Goraya N Wesson DE Acid–base status and progression of chronic kidney disease Curr Opin Nephrol Hypertens 2012 21 552 556 10.1097/MNH.0b013e328356233b 22874469

[B53] Whitescarver SA Ott CE Jackson BA Guthrie GP Jr Kotchen TA Salt-sensitive hypertension: contribution of chloride Science 1984 223 1430 1432 10.1126/science.6322303 6322303

[B54] Kurtz TW Al-Bander HA Jr Morris RC “Salt-sensitive” essential hypertension in men. Is the sodium ion alone important? N Engl J Med 1987 317 1043 1048 10.1056/NEJM198710223171702 3309653

[B55] Shore AC Markandu ND MacGregor GA A randomized crossover study to compare the blood pressure response to sodium loading with and without chloride in patients with essential hypertension J Hypertens 1988 6 613 617 10.1097/00004872-198808000-00003 3183367

[B56] de Solis AJ Gonzales-Pacheco FR Deudero JJ Neria F Albalate M Petkov V Susanibar L Fernandez-Sanchez R Calabia O Ortiz A Caramelo C Alkalinization potentiates vascular calcium deposition in an uremic milieu J Nephrol 2009 22 647 653 19809998

